# Efficacy of Zinc Supplement in Minimal hepatic Encephalopathy: A prospective, Randomized Controlled Study (Zinc-MHE Trial)

**DOI:** 10.31557/APJCP.2021.22.9.2879

**Published:** 2021-09

**Authors:** Rattaya Janyajirawong, Ratha-Korn Vilaichone, Supatsri Sethasine

**Affiliations:** 1 *Division of Gastroenterology and Hepatology, Department of Medicine, Faculty of Medicine, Vajira Hospital, Navamindradhiraj University, Bangkok, Thailand. *; 2 *Excellence Center in Digestive Diseases, Faculty of Medicine and Chulabhorn International College of Medicine (CICM), Thammasat University, Pathumthani, Thailand. *; 3 *Division of Gastroentero-Hepatology, Department of Internal Medicine, Faculty of Medicine, Universitas Airlangga, Surabaya, Indonesia. *

**Keywords:** Zinc supplement, hepatic encephalopathy, quality of life (QoL)

## Abstract

**Background:**

Minimal hepatic encephalopathy (MHE) in patients with cirrhosis of the liver has a negative impact on the quality of daily life by impairing attention, memory and visuomotor coordination, and resulting in cognitive decline. Ammonia is thought to be part of the pathogenesis of hepatic encephalopathy. Zinc is an essential trace element, one of the cofactor enzymes that is essential for the conversion of ammonia to urea.

**Aim:**

To assess the effect of zinc supplementation on psychomotor performance in cirrhotic patients with MHE.

**Methods:**

This prospective, randomized, controlled trial recruited 69 cirrhotic patients (age 18-75 years) diagnosed with MHE by neuropsychometric (NP) tests comprised of the number connection test part A (NCT-A), number connection test part B (NCT-B), serial dot test (SDT), line tracing test (LTT) and digit symbol test (DST). Eligible patients were randomly assigned (1:1) by a computer-based system block of four randomizations to receive 45 mg of elemental zinc or placebo for 12 weeks. The primary endpoint was the absolute change in NP tests from baseline to 12-weeks of zinc supplement compared with placebo. The assessment of changes of the health-related quality of life (HRQOL) using the Short Form survey-36 (SF-36) questionnaire, as well as biochemical parameters including serum ammonia, was also conducted in both groups.

**Results:**

From January to December 2020, 125 eligible cirrhotic patients were diagnosed with liver cirrhosis, of whom 69 (55%) had MHE and were randomly assigned to treatment: 35 patients were assigned to receive 45 mg of elemental zinc and the others 34 patients to receive placebo. Significant improvements in NP tests were established in the zinc supplement group when compared with the placebo group (NCT-A, p = 0.029; NCT-B, p = 0.008; SDT, p = 0.002; DST, p = <0.001). A significant improvement of HRQOL assessed by the SF-36 score was only seen in the zinc group (p<0.001). In the zinc supplement group, not only was an improvement in psychomotor performance reported, but quality of life was also improved, irrespective of baseline zinc level.

**Conclusion:**

Twelve weeks of zinc supplement in cirrhotic patients with MHE not only had a positive effect on psychomotor performance but also improved HRQOL irrespective to baseline zinc level.

## Introduction

The major complications of cirrhosis include hepatic encephalopathy (HE), ascites and bleeding of the varicea, with hepatocellular carcinoma (HCC) representing the end of the spectrum of chronic liver diseases, independent of etiology.

Hepatic encephalopathy, one of the common complications of cirrhosis, manifests in neuropsychiatric problems. Minimal hepatic encephalopathy (MHE) is the reticence form of HE. The prevalence of MHE is approximately 40-60% of the cirrhotic patients (Maldonado-Garza et al., 2011; Rathi et al., 2019; Elgohary et al., 2020; Das et al., 2001).MHE is not detected by clinical and physical examination or laboratory investigation, but is only detected by neuropsychometric (NP) tests or neurophysiological tests (Vilstrup et al., 2014).

For NP tests, the Psychometric Hepatic Encephalopathy Score (PHES) is the sum score achieved from five psychometric tests comprised of the number connection test part A (NCT-A), number connection test part B (NCT-B), digit symbol test (DST), serial dotting test (SDT) and line tracing test (LTT) (Weissenborn, 2015). A diagnosis of MHE is appropriate if the PHES sum scores is less than -4 points (Weissenborn, 2008).

MHE has a negative impact on declines in various functions of patients by impairing work performance, driving capacity and impaired cognitive function (Felipo et al., 2013; García-García et al., 2018; Urios et al., 2017, Bajaj et al., 2020; Agrawal et al., 2015; Bajaj et al.,2008). Furthermore, MHE not only increases the risk of proceeding to overt hepatic encephalopathy but is also associated with the progression of cirrhosis (Ampuero et al., 2018).

Although the pathogenesis of HE is not well clarified, ammonia is an important biochemical substance in the pathogenesis of HE (Shawcross and Jalan, 2005; Ciećko-Michalska et al., 2012). Zinc is an essential cofactor of ornithine transcarbamylase enzymes which enhance the conversion of ammonia to urea and promote glutamine synthetase for the metabolism of ammonia to glutamine in the skeletal muscle. Zinc supplementation can improve health-related quality of life (HRQOL) by decrement of serum ammonia (Marchesini et al., 1996; Katayama,2020). The aim of the study was to assess the effect of zinc supplementation on psychomotor performance in cirrhotic patients with MHE. 

## Materials and Methods

In this prospective, randomized, controlled trial, patients with cirrhosis were recruited at the liver clinic, department of medicine, Vajira hospital in Thailand From January to December 2020. The diagnosis of cirrhosis was based on clinical and laboratory tests, and imaging by ultrasonography or computed tomography of the abdomen. 

*Inclusion criteria*

Age between 18-75 years at time of screening. Diagnosis of cirrhosis was confirmed by liver function test and ultrasound. Eligible cirrhotic patients were screened for an MHE diagnosis by NP tests comprised of five tests including NCT-A, NCT-B, SDT, LTT and DST. Diagnosis of MHE was confirmed if a summation score was less than -4 points (Weissenborn, 2008; Padilla, 2016; Li et al., 2013; Shiha and Mousa, 2019; Ware et al., 2000). 

*NCT-A*

For testing psychomotor speed, there were 25 sequentially numbered circles on a sheet of paper. Patients were timed while drawing a continuous line from the smallest to the largest number. If an error was made, the patient had to correct it without stopping the timer. Each patient was assigned a total time for the time it took to draw a line from number 1 to number 25 inclusive of error correction time. A shorter time to complete the test indicated better performance.

*NCT-B*

For testing psychomotor speed, attention and mental tracking, there were 25 circles, thirteen of which were sequentially numbered from 1 to 13, while the other twelve were labeled with the first 12 characters of the Thai alphabet. Patients had to draw a line which alternated from the lowest remaining number to the earlier remaining alphabet character. If an error was made, the patient had to correct without stopping the timer. The time it took each patient to draw the line correctly was evaluated. A shorter time to complete the test indicated better performance.

*SDT*

For testing psychomotor speed, patients had to draw a dot in the middle of 100 circles then time spent was evaluated. A shorter time to complete the test indicated better performance.

*LTT*

For testing psychomotor speed and visuomotor ability, patients had to draw a continuous line between two parallel lines without intersecting either of the two given parallel lines. Total time spent was evaluated. A shorter time to complete the test indicated better performance.

*DST*

For testing psychomotor speed and attention patients were shown a group squares with a number in each square and a symbol underneath each number. Patients had to remember and add symbols according to the numbers in the given squares. The test result was evaluated by the number of fields a patient could fill in without any false or omitted fields in 90 seconds. A higher score indicated good performance.

All of the cirrhotic patients with MHE self-administered a 36-item Short Form Survey (SF-36) questionnaire in Thai answering questions about their health-related quality of life (HRQOL). The SF-36 consisted of eight aspects of health totaling 35 questions: assessing 10 questions of physical functioning (PF), 4 questions of role physical (RP), 2 questions of bodily pain (BP), 5 questions of perception health (PH), 4 questions of vitality (VT), 2 questions of social functioning (SF), 3 questions of role emotional (RE) and 5 questions of mental health (MH). The 36th question, which asks about change in health in one year, was not included in the scale or summary scores. Score from each aspect were from 0 to 100, higher scores indicated better health (Ware et al., 2000).

*Exclusion criteria*

Patients with a history of overt HE, gastrointestinal bleeding and spontaneous bacterial peritonitis in the past 6 weeks, neurological diseases caused by impaired cognitive function such as epilepsy, Alzheimer’s disease and dementia, recent alcohol consumption within 6 weeks, and medication affecting psychometric competency like benzodiazepines or antidepressant drugs, concurrent with hepatocellular carcinoma or active other malignancy, pregnancy or blindness.

*Randomization and masking*

Eligible patients were randomly assigned to 1:1 ratio to receive oral zinc or placebo. Randomization was performed using a computer-based system, with a permuted block of four. Zinc supplements present as zinc amino acid (bisglycinate) chelate tablets (75 mg per tablet) consist of elemental zinc (15 mg per tablet), supplemented with 45 mg of elemental zinc per day. Zinc and placebo were provided as tablets in identical containers labelled with coded colors. Both physicians and participants were blinded to the treatment assignment. Blood samples were obtained to evaluate complete blood counts, liver function tests, coagulation factors, and zinc and ammonia levels. The Child-Turcotte-Pugh (CTP) and Model for End-stage Liver Disease (MELD) calculated scores were evaluated. Patient follow-up was every four weeks for medical adherence and patients were monitored for adverse events. Extra visits were allowed in the case of any adverse events. At the end of the 12-week treatment period, each patient repeated the NP tests and SF-36 questionnaire. Blood samples for biochemical indexes, including serum ammonia, were also taken at 12 weeks in both groups.

*Outcomes*

The primary outcome was the absolute mean change of NP tests from baseline and after 12 weeks of zinc supplementation compared with placebo. Secondary outcomes were the mean change in HRQOL, serum zinc and ammonia levels, aminotransferases, albumin, platelet count and INR after treatment compared with baseline. When comparing subgroups divided by baseline zinc level, we compared the effect of zinc supplementation on psychomotor tests and quality of life. The safety of zinc was monitored in terms of adverse events. Adverse events were assessed using the Common Terminology Criteria for Adverse Events (version 5.0).

*Statistical analysis*

All continuous variables are expressed as mean ± standard deviation (SD), whereas categorical variables are expressed as absolute values and percentages. To compare between groups, continuous variables were analyzed by the Independent t test and the Wilcoxon-Mann-Whitney test. To compare the mean change (95%CI) between groups in the subgroup analysis, we used the Independent t test or Mann-Whitney test. Data were analyzed by SPSS version 22 and statistical significance was determined at P < 0.05.

Ethical approval was obtained by the Human Research Ethics Committee of Vajira Hospital, Thailand, and the study was conducted according to the good clinical practice guidelines, as well as the Declaration of Helsinki. All data were fully anonymized before they were assessed. The project number for ethical approval was 001-2020. This study was registered in Thai clinical trial registration No 202104233001.

*Sample Size*

As Mousa (2016) reported in his previous work, the mean and SD of the NCT-A and DST between the intervention group (antioxidants/zinc) and the control group (lactulose) after three months of the treatment; for NCT-A, the time at the end of treatment was 56.8±3.07 and 64.3±5.31 sec, respectively (P < 0.001), and for DST, the points at the end of treatment were 12.36±0.87 and 11.58±1.19, respectively (P < 0.006). The minimum sample size after considering the 95%CI and 80% power of the test of this study will be 29 in each group. After adding a 10% attrition rate, at least 33 subjects in each group will participate in the study.

## Results

A total of 157 cirrhotic patients were screened from January to December 2020. Thirty-two patients met the exclusion criteria and were therefore ineligible, while two others declined to participate. One hundred and twenty-five patients underwent NP tests, of whom 69 (55%) patients were diagnosed with MHE and were randomly assigned into two groups: one group (35 patients) received elemental zinc three times a day with a total dose of 45 mg per day for 12 weeks, and the other (34 patients) received placebo for the same duration. After 12 weeks, we repeated the laboratory measurements, NP test and SF-36 questionnaire. During our follow-up visits, three patients were removed from the study due to the COVID-19 pandemic.

In total, 69 MHE patients were enrolled in the study with a mean age of 58.9 ± 8.6 years; fifty-six patients (81%) were male. The etiology of cirrhosis was alcohol (34.8%; n=24) followed by chronic hepatitis C (33.3%; n=23), chronic hepatitis B (18.8%; n=13), non-alcoholic steatohepatitis (8.7%; n=6) and a small number of cases of autoimmune liver disease (4.3%; n=3). Most patients had a compensated Child-Pugh A without ascites. Just over a quarter (27%) of the patients were currently receiving lactulose treatment to prevent clinical hepatic encephalopathy. In patients (n=19) who used lactulose for HE prophylaxis, baseline ammonia levels did not differ from the level in non-lactulose therapy (97.7 ± 55.7 vs. 90.5 ± 61.1 µmol/L, P = 0.65). None of the patients in our study had a history of previous overt hepatic encephalopathy.

There was no difference in the results for NCT-A, NCT-B, SDT and DST tests between the two groups. At baseline, there was a longer delay time for line tracing test in the zinc supplement group (190.6 ± 44.2 vs. 168.12 ± 47.6 sec, p = 0.046). Baseline serum zinc in our MHE patients showed a marginal deficiency (68.6 ± 18.6 mcg/dL) and baseline serum ammonia ranged from 29.1 to 368.8 µmol/L, without any significant difference between the two groups. Neither baseline synthetic function nor baseline SF-36 scores were different between the two groups (p = 0.97 and p = 0.87, respectively) as shown in Table 2.

*Effect of zinc supplementation on NP tests*

After 12 weeks of zinc supplementation, there was an improvement in the performance of NCT-A, NCT-B, and LTT, and there was a statistically significant improvement in SDT (113.97 ± 30.5 vs. 108.32 ± 29.6; p = 0.003) and DST from baseline (18.74 ± 6.4 vs. 20.5 ± 6.3; p=0.011). In comparison with placebo, the mean change in improvement in most tests was significantly for zinc therapy (NCT-A: p = 0.029; NCT-B: p = 0.008, SDT: p = 0.002; DST: p < 0.001). The mean change in reduction of time of LTT after therapy was not significant in comparison between both groups. (Table 3)

*Effect of zinc supplementation on laboratory parameters*

After zinc supplementation, serum zinc increased from 68.47 to 91.21 mcg/dL. Even though we observed that either an increased platelet count, an improvement in INR or an increase in serum albumin were established from baseline compared with 12 weeks in the zinc therapy group (p = 0.037, p = 0.045, p = 0.041; respectively), the mean changes in these parameters were not significant when compared with the mean changes in the placebo group. After twelve weeks of zinc therapy, there was a trend of declining serum ammonia (89.1 ± 61.2 vs. 72.7 ± 27.5; p=0.058). Neither CTP nor MELD showed an improvement after treatment in both groups (Table 3).

*Effect of zinc supplementation on HRQOL*

In terms of the quality of life questionnaire survey, there was a significant improvement in SF-36 score (p<0.001) including each aspect in terms of physical function, social function, mental health, body pain, physical health, role limitations due to emotional problems and vitality after zinc therapy (p = 0.004, p <0.001, p = 0.041, p = 0.002, p = 0.001, p = 0.002, and p = 0.021, respectively). In comparison with placebo, a significant improvement in the mean SF-36 score was seen with zinc supplementation in most domains, with the exceptions of bodily pain and vitality (Table 3).

As the definition of zinc deficiency was a level lower than 60 mcg/dL (Yanagisawa, 2008; Somi et al., 2012), 12 of the 35 (34.3%) patients had baseline zinc deficiencies. In the baseline zinc deficiency group, the ammonia level was no different compared with the other groups (105.3 + 92 vs. 80.9+ 41 µmol/L, p=0.29). According to comparisons between subgroups separated by baseline zinc level, after 12 weeks of zinc therapy, there was no difference in mean change of improvement in NP test or mean change in improvement of SF-36 score, together with nearly all domains of the questionnaire, except for a higher mean change in mental health which was observed in the baseline normal zinc level.

There was no significant difference reported in side effect events in both groups. Three of the 66 (4.5%) patients reported minor adverse events. The adverse events in both groups were nausea in 1 patient (2.9%) in the zinc group and in 1 patient (3.1%) in the placebo group. Symptoms of dizziness were reported in only 1 patient in the zinc therapy group. Dizziness and nausea in our study were clarified as grade 1 severity by definition of Common Terminology Criteria for Adverse Events; all 3 patients were self-limited.

**Table 1:** Baseline Characteristics of Cirrhotic Patients with Minimal Hepatic Encephalopathy (n=69)

	Zinc group (n=35)	Placebo group(n=34)	p-value
Sex			
Female	6 (17.1%)	7 (20.6%)	0.77
Male	29 (82.9%)	27 (79.4%)	0.77
Age	60.06 ± 8.1	57.74 ± 9.1	0.27
BMI, kg/m^2^	24.96 ± 4.8	24.9 ± 4.8	0.96
Education, years	7.09 ± 3.4	8.79 ± 4.1	0.07
Etiology of cirrhosis; n (%)			
Chronic hepatitis C	15 (42.9%)	8 (23.5%)	0.13
Alcohol	11 (31.4%)	13 (38.2%)	0.62
Chronic hepatitis B	6 (17.1%)	7 (20.6%)	0.77
Non-alcoholic steatohepatitis	3 (8.6%)	3 (8.8%)	1
Autoimmune hepatitis	0 (0%)	2 (5.9%)	0.24
Primary biliary cholangitis	0 (0%)	1 (2.9%)	0.5
Present of esophageal varices	9 (25.7%)	4 (11.8%)	0.22
Ascites	1 (2.9%)	1 (2.9%)	1
Current treatment with beta-blocker	8 (22.9%)	2 (5.9%)	0.08
Current treatment with lactulose	10 (28.6%)	9 (26.5%)	1
Child-Pugh score			
A	33 (94.3%)	31 (91.2%)	0.673
B	2 (5.7%)	3 (8.8%)	0.673

Value presented as mean ± SD. and n (%). P-value corresponds to Independent t and Fisher’s exact test.

**Table 2:** Baseline Results of Neuropsychometric Test, SF-36 Scores and Laboratory Parameters

	Zinc group (n=35)	Placebo group (n=34)	p-value
	mean ± SD	mean ± SD	
NCT-A, sec	64.71 ± 19.2	71.5 ± 43.3	0.41
NCT-B, sec	152.91 ± 44.7	136.68 ± 53.0	0.17
SDT, sec	113.97 ± 30.5	105.12 ± 26.6	0.2
LTT, sec	190.6 ± 44.2	168.12 ± 47.6	0.046
DST, point	18.74 ± 6.4	20.82 ± 8.8	0.27
PF	65.86 ± 12.5	65.56 ± 16.9	0.93
SF	77.63 ± 18.8	78.88 ± 20.9	0.79
MH	65.6 ± 16.2	69.53 ± 17.8	0.34
BP	59.83 ± 18.8	63.85 ± 26.8	0.47
PH	52.43 ± 20.1	51.62 ± 18.4	0.86
RP	57.86 ± 40.1	54.41 ± 42.4	0.73
RM	52.17 ± 37.5	52.94 ± 45.1	0.94
VT	57.94 ± 16.2	58.24 ± 18.9	0.95
SF-36	489.31 ± 125.4	495.03 ± 160.1	0.87
ALT, U/L	44.51 ± 28.1	56.03 ± 35.9	0.14
Albumin, g/dL	4.03 ± 0.5	4.03 ± 0.6	0.97
Platelets x 10^3^	161.4 ± 78.3	145.1 ± 71.9	0.37
INR	1.1 ± 0.1	1.18 ± 0.2	0.06
Zinc, mcg/dL	68.47 ± 20.6	68.65 ± 16.7	0.97
Ammonia, µmol/L	89.1 ± 61.2	95.99 ± 58.1	0.63
MELD	9 ± 3.1	9.15 ± 2.5	0.83
CTP	5.17 ± 0.5	5.32 ± 0.6	0.28

Value presented as mean ± SD and n (%) in NP test and laboratory. And presented as median (IQR) in SF-36 test. P-value corresponds to Independent t and Fisher’s exact test. NCT-A, number connection test part A; NCT-B, number connection test part B; DST, digit symbol test; SDT, serial dotting test; LTT, line tracing test; MELD, Model for End-Stage Liver Disease; CTP, Child-Turcotte-Pugh; PF, physical functioning; SF, social functioning; MH, mental health; BP, bodily pain; PH, perception in health; RP, role-physical; RE, role emotional; VT, vitality

**Figure 1:**
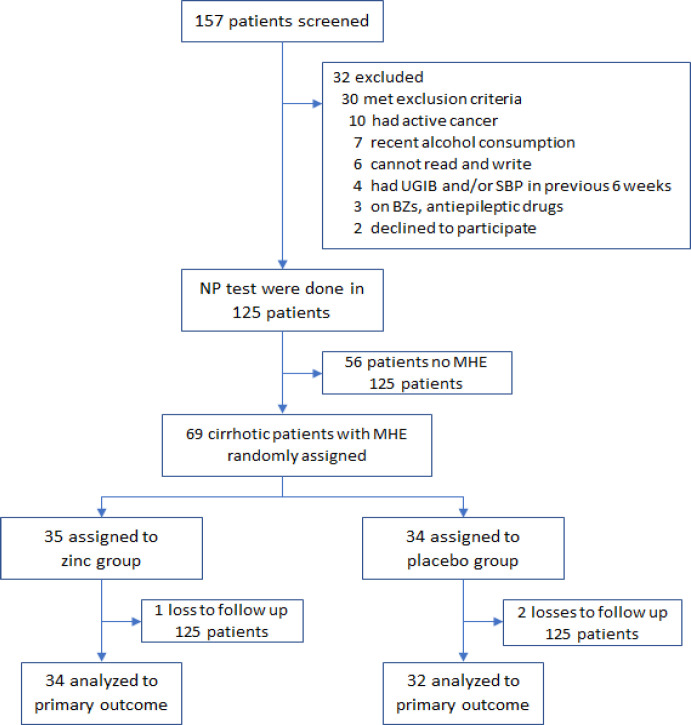
Study Profile

**Figure 2A:**
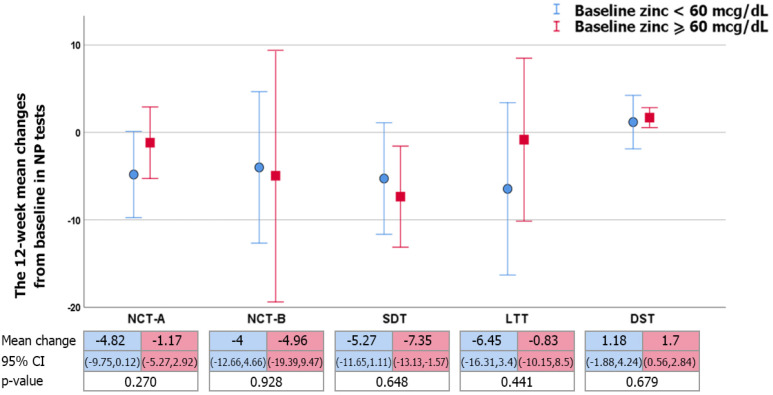
The 12-Week Mean Changes (95%CI) of Neuropsychometric Tests in Zinc Supplement Group, Compared between Baseline Zinc Level < 60 mcg/dL (blue line) and Zinc Level ≥ 60 mcg/dL (red line) Group, as Determined via Independent t Test. Abbreviations: NCT-A, number connection test-A; NCT-B, number connection test-B, SDT, serial dot test; LTT, line tracing test; DST, digit symbol test

**Figure 2B:**
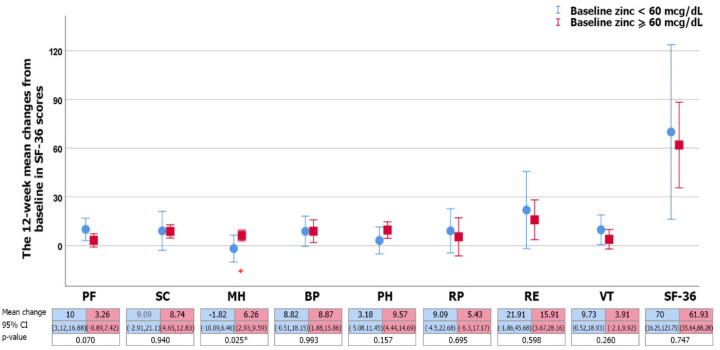
The 12-Week Mean Changes (95%CI) of SF-36 Tests in Zinc Supplement Group, Compared between Baseline Zinc Level < 60 mcg/dL (blue line) and Zinc Level ≥ 60 mcg/dL (red line) Group. *Statistically significant (p < 0.05) comparison between groups as determined via the Independent t test. Abbreviations: PF, physical functioning; SF, social functioning; MH, mental health; BP, bodily pain; PH, perception in health; RP, role-physical; RE, role emotional; VT, vitality

**Table 3:** Neuropsychometric Tests, SF-36 Scores and Laboratory Pparameters at Baseline and after 12-week of Therapy in Both Groups

	Zinc Group			Placebo Group			P3
	Baseline	12 Weeks	P1	Baseline	12 Weeks	P2	
	mean ± SD	mean ± SD		mean ± SD	mean ± SD		
NCT-A, sec	64.71 ± 19.2	62.76 ± 17.3	0.132	71.5 ± 43.3	75.38 ± 42.4	0.113	0.029*
NCT-B, sec	152.91 ± 44.7	148.53 ± 38.8	0.343	136.68 ± 53.0	150.87 ± 51.7	<0.001*	0.008*
SDT, sec	113.97 ± 30.5	108.32 ± 29.6	0.003*	105.12 ± 26.6	110.41 ± 29.8	0.104	0.002*
LTT, sec	190.6 ± 44.2	188.24 ± 41.4	0.436	168.12 ± 47.6	164.63 ± 39.4	0.154	0.855
DST, sec	18.74 ± 6.4	20.5 ± 6.3	0.011*	20.82 ± 8.8	19.09 ± 7.4	0.011	<0.001*
PF	65.86 ± 12.5	71.62 ± 9.9	0.004*	65.56 ± 16.9	63.19 ± 18.5	0.515	0.013*
SF	77.63 ± 18.8	86.79 ± 16.3	<0.001*	78.88 ± 20.9	79.28 ± 23.4	0.669	0.017*
MH	65.6 ± 16.2	68.94 ± 12.2	0.041	69.53 ± 17.8	67 ± 17.8	0.154	0.014*
BP	59.83 ± 18.8	68.79 ± 16.4	0.002	63.85 ± 26.8	65.72 ± 22.3	0.319	0.074
PH	52.43 ± 20.1	59.71 ± 18.1	0.001	51.62 ± 18.4	50 ± 20.8	0.709	0.018*
RP	57.86 ± 40.1	63.97 ± 37	0.13	54.41 ± 42.4	50.78 ± 40.4	0.374	0.096
RM	52.17 ± 37.5	70.59 ± 36.5	0.002	52.94 ± 45.1	59.41 ± 41.3	0.13	0.047*
VT	57.94 ± 16.2	63.09 ± 13.2	0.021	58.24 ± 18.9	58.75 ± 19.3	0.802	0.085
SF-36	489.31 ± 125.4	553.5 ± 110.4	<0.001*	495.03 ± 160.1	494.13 ± 165.9	0.98	<0.001*
ALT, U/L	44.51 ± 28.1	39.85 ± 25.9	0.103	56.03 ± 35.9	57.06 ± 40.2	0.954	0.489
Albumin, g/dL	4.03 ± 0.53	4.2 ± 0.47	0.041*	4.03 ± 0.58	4.23 ± 0.53	0.004*	0.433
Platelets x 10^3^	161.4 ± 78.3	171.6 ± 84.8	0.037*	145.1 ± 71.9	147.6 ± 73.3	0.904	0.089
PT, sec	13.23 ± 1.2	13.21 ± 1.2	0.98	13.73 ± 1.7	13.65 ± 1.7	0.267	0.482
INR	1.1 ± 0.1	1.12 ± 0.1	0.045*	1.18 ± 0.2	1.16 ± 0.1	0.354	0.136
Zinc, mcg/dL	68.47 ± 20.6	91.21 ± 28.5	<0.001*	68.65 ± 16.6	66.25 ± 15.2	0.42	<0.001*
Ammonia, µmol/L	89.1 ± 61.2	72.71 ± 27.5	0.058	95.99 ± 58.1	85.54 ± 44.0	0.129	0.058
CTP score	5.17 ± 0.5	5.03 ± 0.1	0.058	5.32± 0.6	5.19± 0.4	0.057	0.93
MELD	9 ± 3.06	8.74± 1.7	0.561	9.15 ± 2.5	9.5 ± 2.7	0.36	0.288

Value presented as mean ± SD and n (%) in NP test and laboratory. And presented as median (IQR) in SF-36 test. P-value corresponds to Independent t and Fisher’s exact test. NCT-A, number connection test part A; NCT-B, number connection test part B; DST, digit symbol test; SDT, serial dotting test; LTT, line tracing test; MELD, Model for End-Stage Liver Disease; CTP, Child-Turcotte-Pugh; PF, physical functioning; SF, social functioning; MH, mental health; BP, bodily pain; PH, perception in health; RP, role-physical; RE, role emotional; VT, vitality; P1, Mean change before and after zinc therapy; P2, Mean change before and after placebo; P3, Mean change after therapy compared between group

## Discussion

A diagnosis of MHE has often been overlooked by real world practice. MHE is a condition which has a significant detrimental impact on daily quality of life in patients with cirrhosis. The previous prevalence of MHE in cirrhotic patients ranges from 40-60% (Maldonado-Garza et al., 2011; Rathi et al., 2019; Elgohary et al., 2020; Das et al., 2001). In our study, the prevalence of MHE was 55% in compensated cirrhotic patients. 

To the best of our knowledge, zinc deficiency is common in chronic liver disease patients, especially in cirrhosis. Zinc deficiency occurred as the result of several mechanisms such as poor dietary intake, diminished absorption, decreased metabolism, reduction capacity to bind albumin and enhanced protein catabolism (Himoto and Masaki, 2018; Grüngreiff et al., 2016; Kamani and Shaikh, 2018). Zinc deficiency is also a precipitating factor for both covert and overt HE. The prevalence of zinc deficiency is approximately 69% in patients with advanced cirrhosis but the prevalence of this deficiency was not correlated with grading of HE (Loomba et al., 1995; Katayama et al., 2018).The mean zinc level in our MHE patients was 68.6 ± 18.6 mcg/dL, which indicates a marginal level of deficiency. Even though the mean zinc level in our study was higher than the level in previous MHE research, nearly one-third of our patients (21 of 69; 30.4%) had baseline zinc deficiency (Mousa et al., 2016).

The positive impact of zinc supplement as an add-on standard therapy in cirrhotic patients with hepatic encephalopathy was reported in several studies, and can improve the performance of many psychomotor tests, especially the number connection test (Mousa et al., 2016; Takuma et al., 2010; Bresci et al., 1993). Our prospective, randomized, controlled trial study conducted by the use of psychometric hepatic encephalopathy score (PHES) comprised 5 sub-tests, four of which were simple (NCT-A, NCT-B, SDT and DST) and which tested psychomotor speed, attention, and set shifting. The other sub-test tool was assessed for both psychomotor accuracy and additional visuomotor ability examination (LTT). LTT is a test that evaluates a combination of visual performance with movement skills to produce actions. Our results showed a beneficial effect of 12-week zinc monotherapy, as well as a significant improvement in NCT-A, NCT-B, SDT and DST and a trend towards the shortened time for LTT, which currently limits the evaluated by other studies. 

As in aspect of quality of life, previously reported HRQOL score survey in MHE was significantly lower than the HRQOL score in cirrhosis without MHE. A survey revealed that the mean SF-36 score in our MHE patients was much higher than in a previous Chinese Survey in cirrhosis Child A without any grade of encephalopathy (Bo et al., 2007). However, we found an impairment in daily function in our MHE patients following assessment of their HRQOL with the SF-36 general questionnaire. This was a 20% decrease in the score compared with healthy populations (Blake et al., 2000).

Zinc supplementation has a benefit not only by improving of attention but also in cognitive function, including memory. Our HRQOL survey based on the total SF-36 score was significantly improved after zinc monotherapy. When compared with placebo, at least 5 important domains in physical function, social function, perception of health, role emotional and mental health were significantly improved after zinc supplementation. Even though there was an increase in the mean change in bodily pain, limited physical role and vitality in the zinc group, this change was not different in comparison with the other group.

Zinc is a cofactor of ornithine transcarbamylase, which promotes liver ammonia detoxification and the elimination of ammonia from skeletal muscle via the activation of glutamine synthetase. We found baseline serum ammonia to show wide-ranging levels in our MHE patients with a maximal value that was 7-times higher than the upper normal limit. After zinc therapy, blood ammonia levels decreased by approximately 20%; however, there was only a trend towards the non-significant reduction in ammonia levels after zinc supplementation. That was why it was not possible to conclude that the better performance in NP test after zinc Px was caused by a decrease in serum ammonia.

There are many studies of changes in serum ammonia after zinc Px which give conflicting results. Previous studies have shown that zinc supplementation can result in lower ammonia levels in HE grade 1-2 but not in the reduction of recurrent HE (Blake et al., 2000; Riggio et al., 1991; Chavez-Tapia et al., 2013). There was a previous RCT trial of a small number of cirrhotic patients, with varying degrees of HE with hyperammonemia, who were given zinc acetate supplementation at a dosage of 150 mg/day (elemental zinc 45 mg/day) for 3 months, the results of which showed a reduction of serum ammonia but the sample population was too small to clarify a benefit of lowering serum ammonia in the condition of MHE (Katayama et al., 2014). The positive impact of zinc on ammonia level was supported by Hayashi et al., who reported the combination of zinc sulfate 200-600 mg/day (elemental zinc 46-138 mg/day) with branch chain amino acids for 6 months reduced serum ammonia significantly without mentioning the stage of HE (Hayashi et al., 2007).

Most previous HE trials were designed to study the benefit of additional zinc supplementation with lactulose on either serum ammonia, psychomotor test performance or HRQOL (Shiha and Mousa, 2019; Mousa et al., 2016; Takuma et al., 2010; Bresci et al., 1993). On the other hand, in Mousa (2016) studied RCT trials, in particular MHE stage, and reported that three month combination of zinc gluconate 175 mg/day (elemental zinc 25 mg/day) plus antioxidant and lactulose in comparison with only lactulose, which resulted in zinc supplementation with the antioxidant group, can improve NCT-A but produced no difference in the reduction of serum ammonia levels when compared with lactulose therapy alone. Recently, a meta-analysis of the addition of zinc with lactulose in cirrhosis with mild HE (< grade 2) for 3-6 months demonstrated improved NCT tests but did not significantly lower serum ammonia levels when compared with lactulose monotherapy (Shiha and Mousa, 2019). Our study focused on MHE stage and emphasized a positive trend of ammonia reduction together with an improvement in both PHES and HRQOL after 3 months of zinc monotherapy irrespective of the baseline zinc level. Furthermore, whether the continuation of zinc supplementation for longer periods or dose modification may impact serum ammonia and PHES scores in terms of LTT may be of interest in future research.

We observed minor side effects after zinc supplementation in only 2 patients and their symptoms occurred for a few days and self-limited with continuing therapy. The reason why patients maintained good drug compliance was because the form of oral zinc supplement in our study was zinc amino acid (bisglycinate) chelate. It was absorbed differently from other forms of zinc (zinc sulfate, zinc oxide). This zinc bisglycinate consists of one zinc molecule bound to two small molecules of the amino acid glycine. This form was stable in acidic conditions and can be absorbed without decomposition into cationic zinc. In addition, zinc bisglycinate was safer and well-tolerated, with less nausea and vomiting, compared with other zinc formulations (Gandia et al., 2017; DiSilvestro and Swan, 2008). Our study needs a higher dosage and longer duration of zinc supplementation. 

In conclusion, the zinc supplementation in cirrhotic patients with MHE for at least 12 weeks could demonstrate positive effects on psychomotor performance and improved HRQOL irrespective of baseline zinc levels. 

## Author Contribution Statement

Rattaya Janyajirawong and Supatsri Sethasine contributed to the study design. Rattaya Janyajirawong contributed to conduct protocol and data collection. Supatsri Sethasine contributed to data analysis and manuscript writing. Ratha-korn Vilaichone contributed to intensive review of the manuscript. All authors reviewed the final results and approved the final version of the manuscript. 
